# Projection-domain iteration to estimate unreliable measurements

**DOI:** 10.1186/s42492-020-00054-w

**Published:** 2020-07-21

**Authors:** Gengsheng L. Zeng

**Affiliations:** 1 Department of Radiology and Imaging Sciences, University of Utah, 729 Arapeen Drive, Salt Lake City, UT 84108 USA; 2 Department of Computer Science, Utah Valley University, 800 West University Parkway, Orem, UT 84058 USA

**Keywords:** Computed tomography artifacts, Metal artifact reduction, Beam-hardening effects, X-ray computed tomography

## Abstract

Due to the beam-hardening effect of the broad energy spectrum of the X-ray source in computed tomography, the reconstructed images usually suffer from severe artifacts when metallic objects are being imaged. Metal artifact correction methods are usually sophisticated and not practical, especially in some non-medical applications, in which the linear attenuation coefficients are unknown. This paper suggests a simple and effective algorithm to estimate the unreliable measurements. The proposed algorithm is an iterative algorithm, in which the iteration is performed in the projection domain, while the objective function is set up in the image domain. The final image is reconstructed with the conventional filtered backprojection algorithm. The feasibility of the proposed method is verified with airport bags that contain some unknown metals.

## Introduction

In airport bag computed tomography (CT) scans, almost every bag contains some unknown metallic objects. The X-ray tubes in the CT scanners have a broad energy spectrum. The linear attenuation coefficients of the metallic materials vary dramatically within the energy spectrum; this dramatic variation in the linear attenuation coefficients is not properly handled in the reconstruction algorithms. As a result, severe streaking and shadow artifacts appear in the reconstructed images.

Many attempts have been made to battle the metal artifacts. For example, only the projections not affected by the metals are used in an iterative algorithm that also has an edge-preserving prior [1]. Other popular methods are to replace metal affected projections with estimated values. These types of methods are also referred to as projection completion or inpainting [2]. The inpainting methods can use many non-linear image processing techniques such as opening, closing, and segmentation [3]. Inpainting can also be implemented in the Fourier domain, which is essentially lowpass filtering [4]. It is noticed that inpainting can lose spatial resolution; the boundary information from the filtered backprojection (FBP) reconstruction (before inpainting) can be useful [5]. Segmentation in the inpainting methods can be avoided by using histogram deformation [6]. Some authors try to model the polychromatic energy spectrum into the algorithm [7–9]. Using dual-energy CT is able to better synthesize virtual monochromatic images at different photon energy levels, and virtual monochromatic images obtained at high kiloelectron volt levels are known to reduce the effects of beam hardening [8, 9]. Other inpainting methods are also reported in refs. [10–12]. Park et al. [13] proposed a method of correcting metal artifacts due to beam hardening based on the observation that streaking artifacts arise mainly from the geometry of the boundaries of the metallic objects. Using dual energy CT, one can use mathematical models to convert the measurements into synthetic monochromatic measurements. The resultant monochromatic measurements can be used to reconstruct the images with less severe artifacts [14–16]. The mathematical models depend on X-ray source spectrum and metal properties. These properties are not available for unknown objects. Iterative algorithms are popular in image reconstruction when metal objects are involved [17–20]. In ref. [17], an iterative maximum-likelihood polychromatic algorithm was suggested to reconstruct the image. The object’s photoelectric and Compton scatter properties are assumed known. In ref. [18], an iterative polyenergetic statistical algorithm was derived. This algorithm required knowledge of the incident spectrum and knowledge of the distribution of the different types of materials in the object. Reference [19] presented an “alternating minimization” iterative algorithm; the algorithm used prior knowledge of the metal object in the patient, including its pose, shape, and attenuation map. In ref. [20], two methods of estimating the metal affected measurements were compared. It showed that the total variation (TV) inpainting performed better than linear interpolation. In ref. [21], a review was provided for the state-of-art technologies in metal artifact reduction, and the limitations of these technologies were also pointed out. Most recently, machine leaning methods are explored to battle the metal artifacts in CT [22–25]. In ref. [22], an unsupervised deep neural network artifact disentanglement network was proposed to decouple the metal artifacts and the CT images for clinical applications. Reference [23] suggested a conditional generative adversarial network CGAN for data domain sinogram completion. Reference [24] reported a convolutional neural network based metal artifact reduction (CNN-MAR) framework. It was an artifact reduction framework able to distinguish tissue structures from artifacts and fuse the meaningful information to yield a CNN image. By applying the designed tissue processing technique, a good prior was generated to further suppress artifacts. Instead of removing artifacts in the projection domain, ref. [25] proposed a U-net in the image domain. The convolutional layer in the U-net extracted the image and artifact features.

The current metal artifact reduction methods can be roughly divided into two categories. In the first category [2–20], the affected sinogram data is replaced by the estimated data. The estimated data is obtained by using its neighboring measurements and/or by X-ray beam hardening models. These methods do not work well with complicated objects such as airport bags, because one can never have an exact model to predict the beam hardening effects.

In the second category [21–25], the beam hardening effects are “learned” from a large set of measurements with and without metals. The learned model is automatically achieved after the training phase. The results from this category are in general better than those in the first category. However, to gather a large number of representative training images can be overwhelming.

In airport luggage scanning, almost every bag contains metallic objects, and their linear attenuation coefficients are unknown. Therefore, metal artifacts in bag scans are more unpredictable and more difficult to eliminate than in clinical studies, where the metals are usually known. This paper will focus on the airport bag metal artifact reduction.

The TV minimization method was used in ref. [18] to assist inpainting. In ref. [18], the TV norm was evaluated in the projection domain, where the TV method is used as a smoothing filter. In this paper, the TV norm is also used, but the TV norm is evaluated in the image domain instead. Even though the TV norm is evaluated in the image domain, our proposed iteration procedure is carried out in the projection domain. The TV norm is now used as a figure-of-merit for the sinogram consistency. This is the unique feature of our proposed algorithm.

This paper suggests a projection-domain iterative algorithm to estimate the unreliable metal-affected projection measurements. After this step is done, the conventional FBP algorithm is used to reconstruct the final image. Realistic bag scan examples are used to verify the feasibility of the proposed method.

## Methods

### Motivation

The metal artifacts are the beam hardening effects, which are nonlinearly dependent on the metallic materials. These nonlinear effects introduce errors to the line-integral model of the measurements. The line-integral amplitudes are distorted when the integration lines pass through metals. Usually, the distorted line-integral value is smaller than the true value. The distortion is nonlinear and difficult to estimate, because the metallic materials in the objects are unknown.

For a collection of random metallic and non-metallic objects, it is almost impossible to establish a beam hardening model to convert the broad-spectrum measurements into pseudo mono-energy measurements so that the metal artifacts can be removed. On the other hand, machine learning methods do not need the exact mathematic models. Instead, the recent machine leaning methods learn to recognize the metal artifacts and to remove these artifacts. The machine learning methods seem to be more effective and give better results. Inspired by the machine learning methods, this paper gives up on trying to model the beam hardening effects and it focuses on recognizing/reducing the metal artifacts.

A conventional objective function for an iterative algorithm typically has a data-fidelity term and a Bayesian term. This paper proposes an objective function that does not have a data-fidelity term. The proposed objective function only contains one Bayesian term, which the TV normal of the FBP reconstruction. In other words, our objective function is a figure-of-merit. A larger value of this objective function implies more artifacts in the image. Our philosophy of selecting this objective function is as follows. The distortion of the line-integral measurements causes extra structures in the FBP reconstruction. By minimizing the TV-norm of the FBP reconstruction, the extra structures (i.e., artifacts) can be reduced.

In the proposed algorithm, the iteration is only performed for the distorted measurements. The reliable measurements are kept to their original values unaltered.

Our proposed method has the following unique features:

- It does not assume what metals are in the object.
- It does not use the TV method to process the image. It does not use interpolation methods to estimate the metal-affected projection values. It uses the TV objective function to tell us how the metal-affected projection values should be corrected.
- It does not need other good and similar images to train or assist. It does not even use the neighboring projection values to estimate and replace the bad projection values.
- It is not an iterative image reconstruction method, but it is rather an iterative bad projection value replacement method.
- It does not assume any prior knowledge of the object and does not segment the image into some known values.

### Algorithm development

Let *f* be the FBP reconstruction. The image *f* is represented in a two-dimensional array and *f*_*i,j*_ is its pixel value at the *i*th row and *j*th column. The TV norm of *f* is defined as

1 $$ T={\sum}_{i,j}\sqrt{{\left({f}_{i,j}-{f}_{i,j+1}\right)}^2+{\left({f}_{i,j}-{f}_{i+1,j}\right)}^2}, $$

When the quantity under the square root in Equation (1) is positive, the partial derivative of *T* with respect to pixel (*i, j*) is readily calculated as [26]

2 $$ {\displaystyle \begin{array}{c}{U}_{i,j}=\frac{\partial T}{\partial {f}_{i,j}}\\ {}=\frac{\left({f}_{i,j}-{f}_{i,j+1}\right)+\left({f}_{i,j}-{f}_{i+1,j}\right)}{\sqrt{{\left({f}_{i,j}-{f}_{i,j+1}\right)}^2+{\left({f}_{i,j}-{f}_{i+1,j}\right)}^2}}\\ {}+\frac{f_{i,j}-{f}_{i,j-1}}{\sqrt{{\left({f}_{i,j-1}-{f}_{i,j}\right)}^2+{\left({f}_{i,j-1}-{f}_{i+1,j-1}\right)}^2}}\\ {}+\frac{f_{i,j}-{f}_{i-1,j}}{\sqrt{{\left({f}_{i-1,j}-{f}_{i-1,j+1}\right)}^2+{\left({f}_{i-1,j}-{f}_{i,j}\right)}^2}}\end{array}} $$

When the quantity under the square root is zero, the quantity already reaches its minimum and the penalty function is no longer needed. In this situation, it is safe to set the derivative to zero. One easy way to handle this situation in an actual implementation of Equation (2) is to add a very small positive constant *ɛ*, say, 10^−8^, under the square roots in all denominators. Thus Equation (2) becomes

3 $$ {U}_{i,j}\approx \frac{\left({f}_{i,j}-{f}_{i,j+1}\right)+\left({f}_{i,j}-{f}_{i+1,j}\right)}{\sqrt{{\left({f}_{i,j}-{f}_{i,j+1}\right)}^2+{\left({f}_{i,j}-{f}_{i+1,j}\right)}^2+\varepsilon }}+\frac{f_{i,j}-{f}_{i,j-1}}{\sqrt{{\left({f}_{i,j-1}-{f}_{i,j}\right)}^2+{\left({f}_{i,j-1}-{f}_{i+1,j-1}\right)}^2+\varepsilon }}+\frac{f_{i,j}-{f}_{i-1,j}}{\sqrt{{\left({f}_{i-1,j}-{f}_{i-1,j+1}\right)}^2+{\left({f}_{i-1,j}-{f}_{i,j}\right)}^2+\varepsilon }} $$

Thus, an iterative gradient descent algorithm to minimize the objective function *T* defined in Equation (1) is

4 $$ {f}_{i,j}^{new}={f}_{i,j}^{old}-\lambda {U}_{i,j} $$

Equation (4) is not useful, because it will make *f* converge to a constant (i.e., a flat image with *U*_*i*, *j*_ = 0). We will now make some modifications to Equation (4) so that it can be useful for our purposes.

The first modification to Equation (4) is taking the Radon transform on both sides of Equation (4), obtaining

5 $$ {p}_{t,\theta}^{new}={p}_{t,\theta}^{old}-\lambda \mathfrak{R}\left\{{U}_{i,j}\right\} $$

where λ is the relaxation parameter chosen to be 0.01 in this paper, ℜ is the Radon transform operator, and ${p}_{t,\theta }=\mathfrak{R}\left\{f\right\}$ with (*t*, *θ*) being the measurement space coordinates. In fact, Equation (5) is not useful, like Equation (4), only driving *U*_*i,j*_ to 0.

The second modification is to multiply a mask function on both sides of Equation (5). In order to find this mask function, we first use the FBP algorithm to reconstruct an initial image, which may contain lots of metal artifacts. Select a threshold value (for example, 1/3 of the maximum image value), and use this value to segment the FBP reconstruction to create a metal object image *f*_*metal*_. The mask, denoted as *mask*_*metal*_, is a function in the projection-domain and is based on the Radon transform of *f*_*metal*_. The mask function *mask*_*metal*_ is 1 at the location that the Radon transform of *f*_*metal*_ is positive and is 0 at the location that the Radon transform of *f*_*metal*_ is 0. The mask function restricts Equation (5) only on the region when *mask*_*metal*_ is 1. Thus, Equation (5) is further modified as

6 $$ {p}_{t,\theta\ (metal)}^{new}={p}_{t,\theta\ (metal)}^{old}-\lambda \times {mask}_{metal}\times \mathfrak{R}\left\{{U}_{i,j}\right\} $$

Equation (6) only updates the line-integral measurements that the projection rays *p*_*t,θ(metal)*_ touch the metal objects.

Equation (6) is still not satisfactory, because it will smooth out all metallic objects while minimizing the TV norm (Equation1). In order to keep the metallic objects in the image, the image-domain masking function *f*_*metal*_ can be used to hide the metals in TV gradient image *U*_*i,j*_, that is, replacing *U*_*i,j*_ by (*U*_*i,j*_) × (1- *f*_*metal*_). At a pixel with metal, (1- *f*_*metal*_) = 0. At a pixel without metal, (1- *f*_*metal*_) = 1. The final algorithm proposed in the paper is, therefore, expressed as

7 $$ {p}_{t,\theta (metal)}^{new}={p}_{t,\theta (metal)}^{old}-\lambda \times {mask}_{metal}\times \mathfrak{R}\left\{{U}_{i,j}\times \left(1-{f}_{metal}\right)\right\} $$

The proposed algorithm can be implemented in the following steps:

1. Obtain a raw FBP reconstruction.
2. Use a threshold to segment a metal image, *f*_*metal*_, from the raw FBP reconstruction.
3. Set the raw FBP reconstruction as the initial image and perform iterative updates for the unreliable measurements *p*_*t*, *θ*(*metal*)_ according to Equation (7).
4. Obtain the final FBP reconstruction with the measurements, the unreliable portions of them have been revised by step (3).

### Airport bag experiments

The original projections of airport bags were acquired with an Imatron C300 clinical CT scanner, which was a fifth generation CT scanner based on a scanning electron beam X-ray source manufactured in the late 1990s. The original projection data was rebinned and downsized for our reconstruction in this paper. The number of views was 180 over 180°. The number of channels (i.e., the detection bin at each view) was 597. The projections used the parallel-beam imaging geometry. The reconstructed image size was 420 × 420.

## Results

**Fig. 1:**
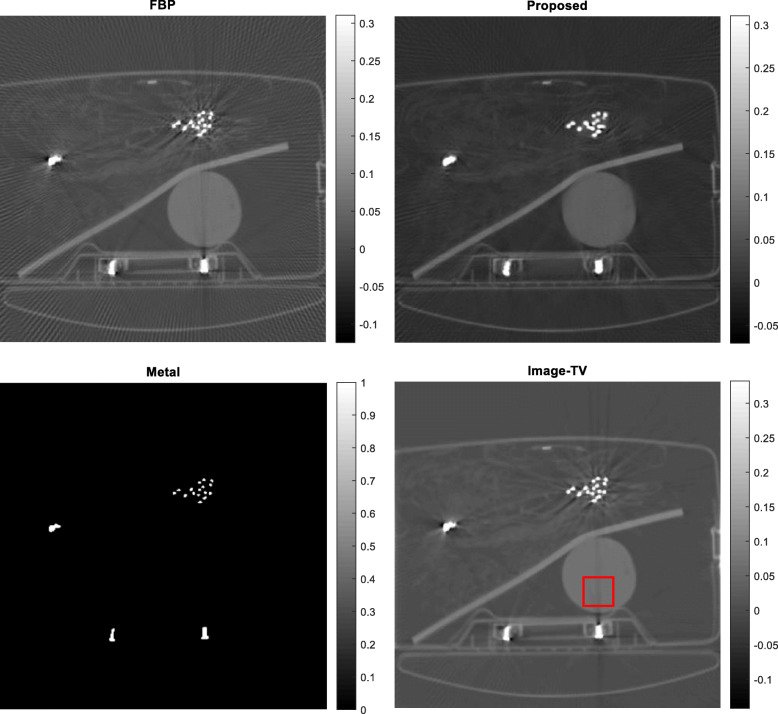
Airport bag #1. Upper row, Left: conventional FBP reconstruction; Lower row, Left: metal segmentation; Upper row, Right: FBP reconstruction after proposed damaged value recovery; Lower row, Right: image-domain TV reconstruction. The small red square indicates the ROI

**Fig. 2:**
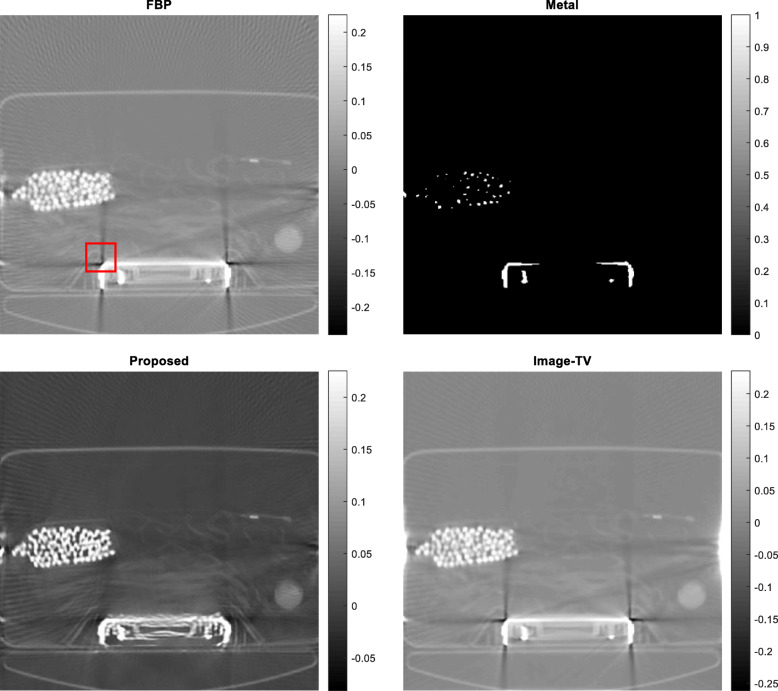
Airport bag #2. Upper row, Left: conventional FBP reconstruction; Upper row, Right: metal segmentation; Lower row, Left: FBP reconstruction after proposed damaged value recovery; Lower, Right: image-domain TV reconstruction. The small red square indicates the ROI

**Fig. 3:**
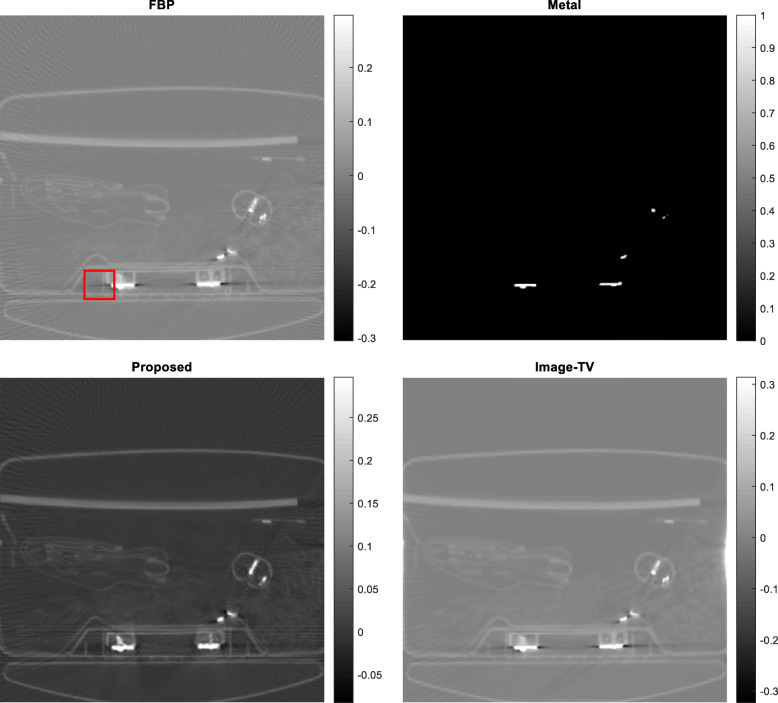
Airport bag #3. Upper row, Left: conventional FBP reconstruction; Upper row, Right: metal segmentation; Lower row, Left: FBP reconstruction after proposed damaged value recovery; Lower, Right: image-domain TV reconstruction. The small red square indicates the ROI

**Fig. 4:**
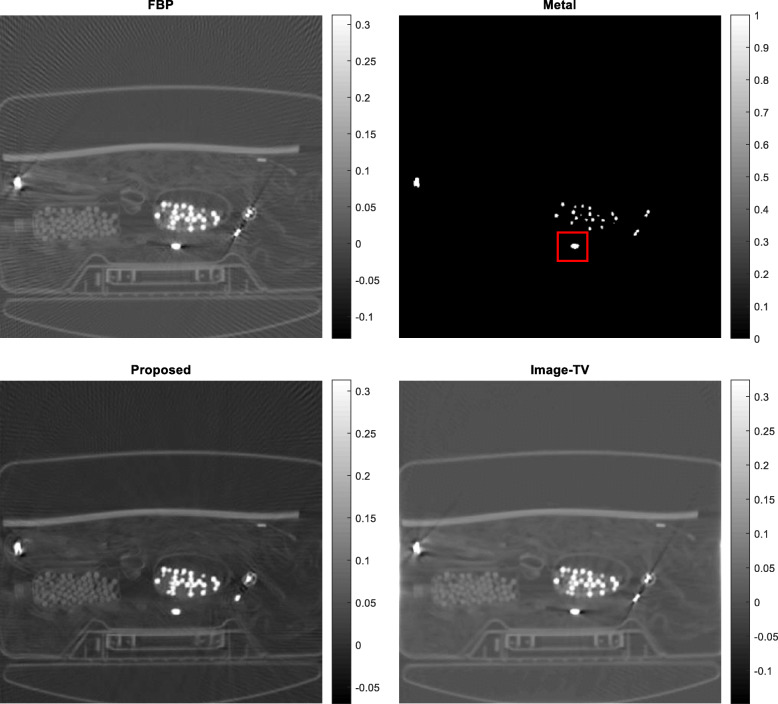
Airport bag #4. Upper row, Left: conventional FBP reconstruction; Upper row, Right: metal segmentation; Lower row, Left: FBP reconstruction after proposed damaged value recovery; Lower, Right: image-domain TV reconstruction. The small red square indicates the ROI

**Fig. 5:**
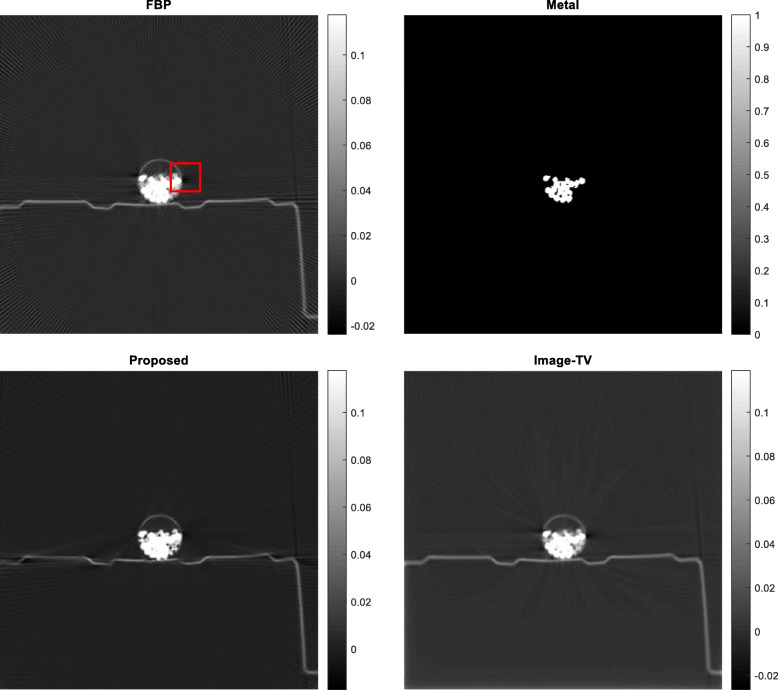
Airport bag #5. Upper row, Left: conventional FBP reconstruction; Upper row, Right: metal segmentation; Lower row, Left: FBP reconstruction after proposed damaged value recovery; Lower, Right: image-domain TV reconstruction. The small red square indicates the ROI

Five airport-bag examples are presented here to illustrate the feasibility of the proposed algorithm. For each example, the following 5 images are shown: (1) the raw FBP reconstruction, (2) the segmented metal image *f*_*metal*_, (3) the projection-domain mask *mask*_*metal*_, (4) the final FBP reconstruction using the restored projections, and (5) the reconstructed image using the image-domain TV iterative algorithm. The image-domain TV iterative algorithm is similar to Equation (4), by adding a data fidelity term. The relaxation parameter for the data fidelity term was 0.001 and the relaxation parameter for the TV constraint term was 0.0005. The number of iterations was 400 for both the proposed projection-domain iteration algorithm and for the image-domain iterative TV algorithm. The reconstructions are displayed from the minimum image value to 0.3 times the maximum image value. The results from these five examples are shown in Figs. 1, 2, 3, 4 and 5, respectively.

**Fig. 6:**
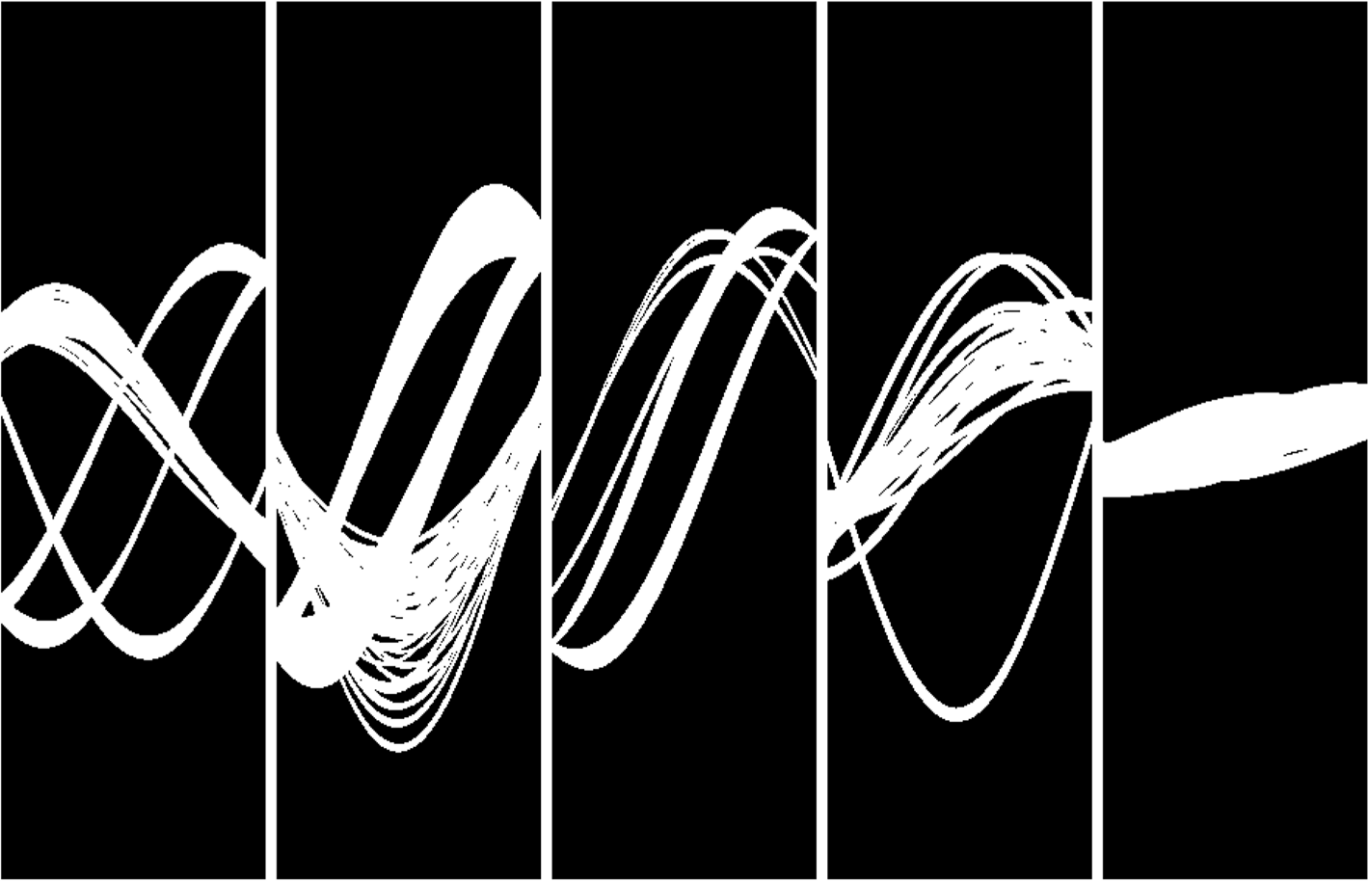
From left to right: Projection masks for measurements of bags 1 to 5, respectively. These are binary images with values 0 (black) and 1 (white). The white regions indicate the projection values that are adjusted by the proposed algorithm. The black regions indicate the projection values that are kept unchanged

Figure 6 shows the projection masks for the measurements of bags 1 to 5, respectively. These are binary images with values 0 (black) and 1 (white). The white regions indicate the projection values that are adjusted by the proposed algorithm. The black regions indicate the projection values that are kept unchanged.

In this section, three methods are compared in terms of their performance on correcting metal induced errors. These three methods are: the conventional FBP algorithm, the TV-minimization algorithm with image-domain update, and the proposed TV-minimization algorithm with projection-domain update. For the five airport bags, the reconstructed images using these three methods are shown in Figs. 1, 2, 3, 4 and 5, respectively.

The metal object induced artifacts appear as dark undershoots around the bright metal objects. Visual assessments indicate that the conventional FBP algorithm gives the most severe artifacts, the image-domain-update TV algorithm somewhat reduces the artefacts with worsened spatial resolution, and the proposed algorithm is most effective in metal artifact reduction.

Numerical evaluation of the metal artifact reduction is by measuring the minimum image pixel value in the dark undershoot region. Firstly, an undershoot region-of-interest (ROI) is identified visually. The ROI is a 40 × 40 square region. Secondly, the minimum value in this ROI is searched. This minimum value serves as the figure-of-merit. A smaller minimum value indicates a more severe artifact. A smaller value is a value closer to the negative infinite. The numerical results are summarized in Table 1, from which the proposed method performs the best among the three methods.

**Table 1:** Artifact severity comparison in an ROI

Bag	Conventional FBP method	Image-domain update TV method	Projection-domain update TV method (proposed)
1	0.2491	0.2625	0.2848
2	−0.2616	− 0.2491	0.0983
3	−0.3233	−0.314	0.0971
4	−0.1485	−0.1104	0.148
5	−0.0125	0.0076	0.0466

A smaller value indicates a more severe artifact. A larger number indicates better performance. Do not compare between bags, because they contain different objects

**Fig. 7:**
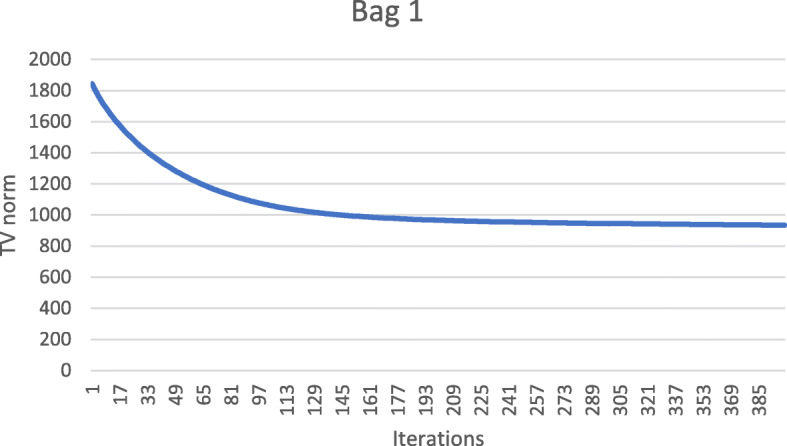
Airport bag #1. TV norm of the image as a function of the iteration number

**Fig. 8:**
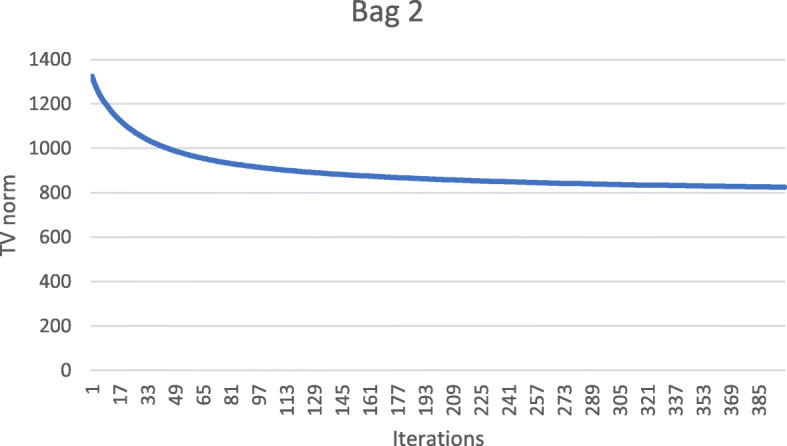
Airport bag #2. TV norm of the image as a function of the iteration number

**Fig. 9:**
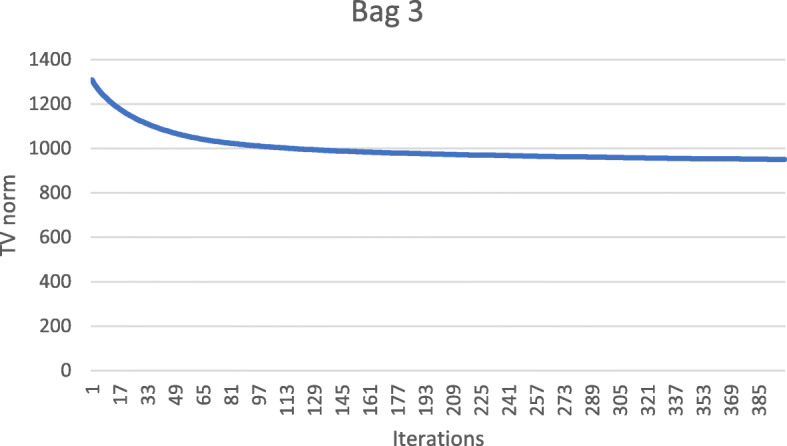
Airport bag #3. TV norm of the image as a function of the iteration number

**Fig. 10:**
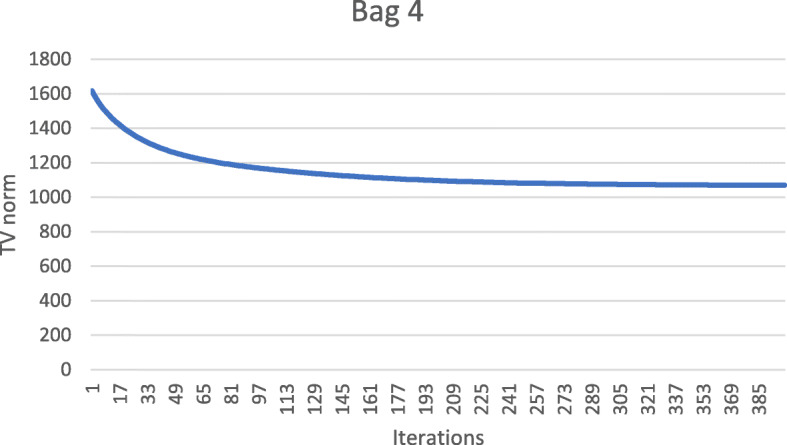
Airport bag #4. TV norm of the image as a function of the iteration number

**Fig. 11:**
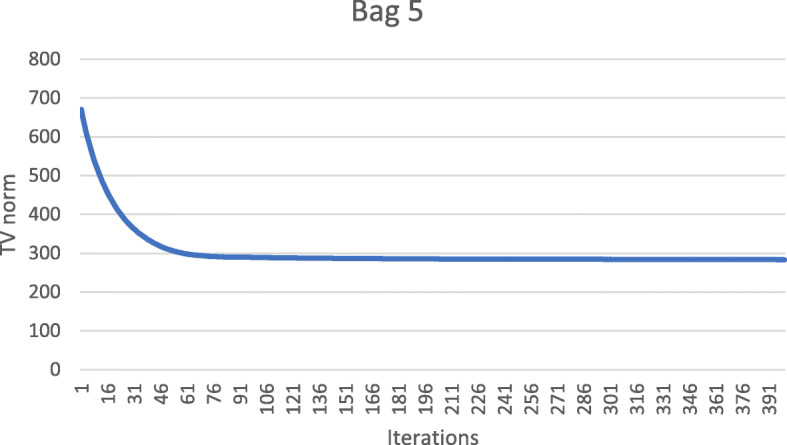
Airport bag #5. TV norm of the image as a function of the iteration number

These numerical results also imply that the metal streaking artifacts can be reflected by the image TV norm. Figures 7, 8, 9, 10 and 11 show the convergence curves of the image TV norm versus the iteration number for the proposed algorithm for the five airport bags, respectively.

## Conclusions

The proposed projection-domain iterative algorithm minimizes the image-domain TV norm of the FBP reconstruction. This algorithm does not use any models for the unreliable projections. Therefore, this algorithm can be applied to many applications in addition to removing beam-hardening artifacts. The proposed method may be effective in applications where most of the measurements are with excellent quality and few measurements are severely damaged. The few measurements cause some severe streaking artifacts. The proposed method may not be effective in applications where there are too many unreliable measurements. The proposed method may not be effective in image de-noising, for example, in acoustic imaging [27] for speckle reduction, because the error sources are distributed to all measurements.

This paper has achieved the goal of effectively reduce the metal artifacts without any prior knowledge of the X-ray source spectrum and the metal properties. The main restriction of the proposed method is that most measured projections are accurate, and only a small portion of the measurements are severely damaged. Our future research includes investigation of using more constraints so that we can handle the situations, where more measurements are severely damaged.

## Data Availability

Not applicable.

## Abbreviations

FBP: Filtered backprojection
ROI: Region-of-interest
TV: Total variation
CT: Computed tomography
CNN: Convolutional neural network
